# Immune responses to an early lytic cytomegalovirus antigen in systemic lupus erythematosus patients: T-cell responses, cytokine secretions and antibody status

**DOI:** 10.1371/journal.pone.0193244

**Published:** 2018-03-02

**Authors:** Anette Holck Draborg, Niclas Stefan Rasmussen, Janni Lisander Larsen, Charlotte Sværke Jørgensen, Noreen Sandhu, Kristin Skogstrand, Søren Jacobsen, Gunnar Houen

**Affiliations:** 1 Department of Autoimmunology and Biomarkers, Statens Serum Institut, Artillerivej 5, Copenhagen, Denmark; 2 Copenhagen Lupus & Vasculitis Clinic, Centre for Rheumatology and Spine Disease, Rigshospitalet, Copenhagen University Hospital, Blegdamsvej 9, Copenhagen, Denmark; 3 Department of Virus & Microbiological Special Diagnostics, Statens Serum Institut, Artillerivej 5, Copenhagen, Denmark; 4 Center for Neonatal Screening, Department of Congenital Disorders, Statens Serum Institut, Artillerivej 5, Copenhagen, Denmark; Instituto Nacional de Ciencias Medicas y Nutricion Salvador Zubiran, MEXICO

## Abstract

We investigated immune responses to a lytic cytomegalovirus antigen (CMVpp52), and to a lytic human herpes virus (HHV) 6 antigen (HHV6p41), in systemic lupus erythematosus (SLE) patients and healthy controls (HCs), in order to clarify if the previously established impaired responses to Epstein-Barr virus (EBV) in SLE patients is a general defect in their responses against (all) HHVs. Multiplex Luminex technology results showed a normal induction of five quantified cytokines (interferon γ, interleukin(IL)12, IL17, IL10, and tumor necrosis factor α) in SLE patients compared to HCs upon stimulation with CMVpp52 and HHV6p41. However, flow cytometric results showed a reduced upregulation of the activation marker CD69 on T-cells from SLE patients (n = 17) compared to HCs (n = 17) upon stimulation with CMVpp52, indicating limited or defective CMVpp52-specific T-cells and/or poor antigen-presentation in SLE patients, and thereby possibly decreased control of the CMV infection. In conclusion, the dysfunctional immune response against EBV previously established in SLE patients does not seem to apply to the same degree regarding the immune responses against CMV or HHV6. Results designate that the main contributing HHV agent in development or exacerbation of SLE (in genetically predisposed individuals) is the previously determined uncontrolled EBV infection, and to a lesser extent CMV infection, and probably with no involvement of HHV6 infection.

## Introduction

Systemic lupus erythematosus (SLE) is an autoimmune disease that typically presents in women. It is characterized by heterogeneous clinical manifestations, including production of various autoantibodies and disease flares, alternating with remissions. The etiology behind development of SLE is complex and involves both genetic predispositions and environmental factors, particularly infections with human herpes viruses (HHVs). [1–8]

HHVs comprise eight viruses including Epstein-Barr virus (EBV, HHV4), cytomegalovirus (CMV, HHV5), and human herpes virus 6 (HHV6). They are dsDNA viruses and ubiquitous infectious agents infecting the majority of the world’s population. They have a latent state, from which they occasionally reactivate and establish a productive cycle [9–11]. The tropism varies greatly among the viruses. Latent infections are established mainly in resting B-cells regarding EBV, and mainly in monocytes and hematopoietic stem cells regarding CMV, and in monocytes regarding HHV6 [12–14]. The immune system is capable of keeping a tight control of the HHV infections in immune competent individuals, and cell-mediated immunity is fundamental in this regard.

The association between SLE and EBV infection is by far the most studied and shows reduced control of the EBV infection, with elevated seroprevalence and elevated titers of EBV antibodies against lytic cycle antigens, decreased T-cell responses against EBV, and increased viral load in SLE patients compared to healthy controls (HCs) [8, 15–19].

The association between SLE and CMV infection has also previously been investigated and has shown increased percentages of SLE patients positive for CMV DNA [20, 21]. Studies on CMV-directed antibodies in SLE patients have shown increased titers of IgG and IgA antibodies against CMVpp52, which is an early lytic cycle antigen necessary for lytic viral replication [22]. Furthermore, elevated percentages of IgM and IgG antibodies to unspecified CMV antigens have been observed in SLE patients compared to HCs [20, 23–25]. Using HLA/CMVpp65-peptide tetramers, Larsen et al. showed a normal amount of CMVpp65-specific CD8+ T-cells in SLE patients with normal cytokine responses to CMV stimulation and no increased viral load [26]. Kang et al. merely showed a tendency of a reduced CMV-directed T-cell response, when whole blood samples were stimulated with CMV antigens [27].

Only a few studies have examined HHV6 infection in SLE patients. Rasmussen et al. showed no difference between SLE patients and HCs in antibody (IgM, IgG and IgA) titres against HHV6p41 (which is a HHV6 early lytic antigen) [22]. However, two other studies have shown an association between SLE and active HHV6 infection [28, 29].

The current study is a continuation of our previously published results on EBV-directed immune responses in SLE patients [18, 30] but with focus on CMV and HHV6. We sought to determine if our previously observed results on reduced T-cell response and cytokine response pattern upon EBV antigens stimulation [18, 30] is a general defect in the immune responses against HHVs in SLE patients. Thus, we investigated the T-cell response to CMV and the cytokine response pattern induced by stimulation with CMV and HHV6 antigens. CMVpp52 and HHV6p41 were chosen as stimulatory antigens as they are lytic cycle antigens and functional homologues to the previously investigated stimulatory agent EBV-EA/D (EBV early antigen diffuse), and also the antibody response against these two antigens have previously been determined [22]. In a 4-color flow cytometric assay the T-cell response to CMVpp52 was measured using CD69 as an early marker for activation and interferon(IFN)γ-production in the individual T-cells as a marker for the anti-viral response. These results on CMVpp52-responding T-cells were compared with the CMVpp52 antibody status. Furthermore, in a separate experimental setup, T-cell-related cytokines (IFNγ, interleukin(IL)12, IL17), and also, one inflammatory (tumor necrosis factor(TNF)α) and one anti-inflammatory (IL10) cytokine induced upon stimulation with CMVpp52 and HHV6p41 were measured by Luminex technology.

## Materials and methods

### SLE patients and healthy controls

Two different setups were applied in the current study using two different SLE patient/HC cohorts. In both cases were heparinized whole blood samples from SLE patients collected at Copenhagen Lupus & Vasculitis Clinic, Centre for Rheumatology and Spine Disease, Rigshospitalet, Copenhagen University Hospital, Copenhagen, Denmark, and blood samples from apparently HCs were collected from volunteers at Statens Serum Institut, Copenhagen, Denmark. All patients fulfilled internationally accepted classification criteria for SLE [31].

During flow cytometric analyses of EBV-induced activation of T-cells in SLE patients [18], the CMVpp52-induced T-cell response was additionally investigated by flow cytometry in a subset of the cohort comprising 17 age- and sex-matched pairs of the included SLE patients and HCs. The characteristics of these 17 SLE/HC-pairs are outlined in Table 1, left column.

**Table 1:** Characteristics of SLE patients and HCs in the two studies.

		Study: Flow cytometric T-cell analysis	Study: Cytokine quantification by luminex
		SLE patients cohort 1	SLE patients cohort 2
No. of individuals		17	17
Mean age (years) (range)		40.7 (21–81)	42,6 (23–61)
Females		94%	100%
Disease manifestations:			
	Nephritis	29%	12%
	Vasculitis	0%	0%
	Arthritis	29%	6%
	Rash	18%	18%
	Alopecia	0%	0%
	Myositis	0%	0%
	Mucosal ulcers	0%	0%
	Serositis	6%	6%
	Leucopenia	12%	0%
	Thrombocytopenia	18%	0%
	Visual disturbance	0%	0%
	Fever	18%	0%
Mean SLEDAI score (range)		6.7 (0–22)	3.2 (0–12)
dsDNA antibody positive		47%	65%
Rheumafactor positive			
	IgA	12%	29%
	IgM	0%	24%
Low C3 or C4 level		71%	35%
Mean C-reactive protein (mg/L) (range)		5.7 (1–22)	6.4 (1–38)
Medication:			
	Prednisolone (median dose, mg)	65% (5)	24% (0)
	Azathioprine (median dose, mg)	29% (0)	18% (0)
	Mycophenolate mofetil (median dose, mg)	24% (0)	18% (0)
	Methotrexate (median dose, mg)	12% (0)	6% (0)
	Hydroxychloroquine (median dose, mg)	59% (200)	47% (0)
	Anticoagulant	35%	47%
	Antihypertension	24%	35%
		**HCs cohort 1**	**HCs cohort 2**
No. of individuals		17	17
Mean age (years) (range)		37.3 (25–59)	42.9 (28–64)
Females		94%	100%

In addition, another cohort of 22 included SLE patients and age- and sex-matched HCs (with no overlap to the 17 SLE patient/HC-pairs investigated above) was investigated in the current study for CMVpp52-induced cytokine induction (and HHV6p41-induced cytokine induction in a subset of eight SLE patient/HC-pairs). Five of the included 22 SLE patients were found to suffer from lymphopenia (<1.00*10^9^/L) and were excluded in order to assess the issue without an effect on results due to low levels of T-cells. The characteristics of these 17 investigated SLE/HC-pairs are shown in Table 1, right column.

### Ethics statement

The studies were approved by the Scientific Ethical Committee of the Capital Region of Denmark (no. HA-2007-0114 and no. H-15009640, for the two cohorts, respectively). Written informed consent was obtained from all participants before inclusion.

### Ex vivo stimulation of whole blood samples with antigens

Two *ex vivo* stimulation setups were employed during the studies. One for flow cytometric T-cell analyses and another for quantification of antigen-induced cytokines by Luminex technology.

The *ex vivo* stimulations of whole blood samples for flow cytometric T-cell analyses were made simultaneously with the stimulations in a previous study [18]. Heparinized whole blood samples were incubated at 37°C for 24 hours with recombinant CMVpp52 (5 μg/ml, *Escherichia coli* (*E-coli*)-derived, CMV-214, Prospec Protein Specialist, Ness-Ziona, Israel) and with staphylococcal enterotoxin B (SEB) and phosphate-buffered saline (PBS) as positive and negative controls, respectively. Eight hours subsequent to stimulation, Brefeldin A (10 μg/ml, Sigma-Aldrich, St- Louis, Missouri, USA) was added to inhibit cytokine secretion from activated cells. Stimulation was terminated after 24 hours by addition of 20 mM EDTA (100 μl/ml), and samples immediately employed for staining and subsequent flow cytometric T-cell analysis (see below).

The *ex vivo* stimulations of whole blood samples for quantification of induced cytokines by Luminex technology were executed correspondingly as above, but without the addition of the secretion inhibitor. In addition, stimulation was also performed using recombinant HHV6p41 (5 μg/ml, *E-coli*-derived MyBiosource, San Diego, CA, USA) and also an extra negative control was included by stimulation with *E*.*coli*-derived β-galactosidase (5 μg/ml, Sigma-Aldrich, St. Louis Missouri, USA). The latter to ensure that the stimulation of cytokine production was not due to the fact that the CMVpp52 and HHV6p41 antigens are produced in *E*.*coli* and possibly contaminated with lipopolysaccharide (LPS). Subsequent to stimulation, 100 μl stimulated whole blood was spotted, dried and stored at -20°C as dried blood spot samples (DBSS) on filter paper until all patients and HCs were included in the study and then used for cytokine quantification by Luminex technology (see below).

To facilitate comparability between results on SLE patients and HCs, each age- and sex-matched SLE/HC-pair had the same lag-time before stimulation in both experimental setups. The lag-time was defined as the period of time from blood collection until beginning of stimulation. In setup 1 with stimulations for flow cytometric T-cell analysis, the lag-time ranged between 44 and 81 minutes, and in setup 2 with stimulations for quantification of induced cytokines by Luminex technology, the lag-time ranged between 50 and 171 minutes.

### Flow cytometric T-cell analysis

The staining and detection of activated (CD69-positive) and IFNγ-producing T-cells in the stimulated whole blood samples (here with CMVpp52, SEB and PBS) was described previously [18]. In short, erythrocytes were initially lysed and next the lymphocytes were permeabilised. After washing, the lymphocytes were stained with a mixture of allophycocyanin (APC)-conjugated anti-CD3, cyanine 5.5 peridinin chlorophyll (PerCP-Cy5.5)-conjugated anti-CD8, phycoerythrin (PE)-conjugated anti-CD69, and fluorescein isothiocyanate (FITC)-conjugated anti-IFNγ (20 μl in total, BD Biosciences, San Jose, CA, USA). In addition, another SEB-stimulated sample from each individual was stained with an isotype-specific control antibody mix (anti-CD3- APC, anti-CD8-PerCP-Cy5.5, IgG_1_-PE, and IgG_2a_-FITC, BD Biosciences, San Jose, CA, USA) to assess the staining and also the non-specific staining and to check for autofluorescence. After staining, washing and fixation, the flow cytometric analyses were done on a FACScalibur flow cytometer and CELLQuest software (BD Biosciences, San Jose, CA, USA). CaliBRITE beads (BD Biosciences, San Jose, CA, USA) were used to accomplish fluorescence compensations. Data was analyzed by the use of FLOW JO software (Tree Star, San Carlos, CA, USA).

Data for a large number of CD3-positive events (100000) was acquired on the flow cytometer in order to ensure that even low percentages of activated cells account for a considerable number of cells.

**Fig 1:**
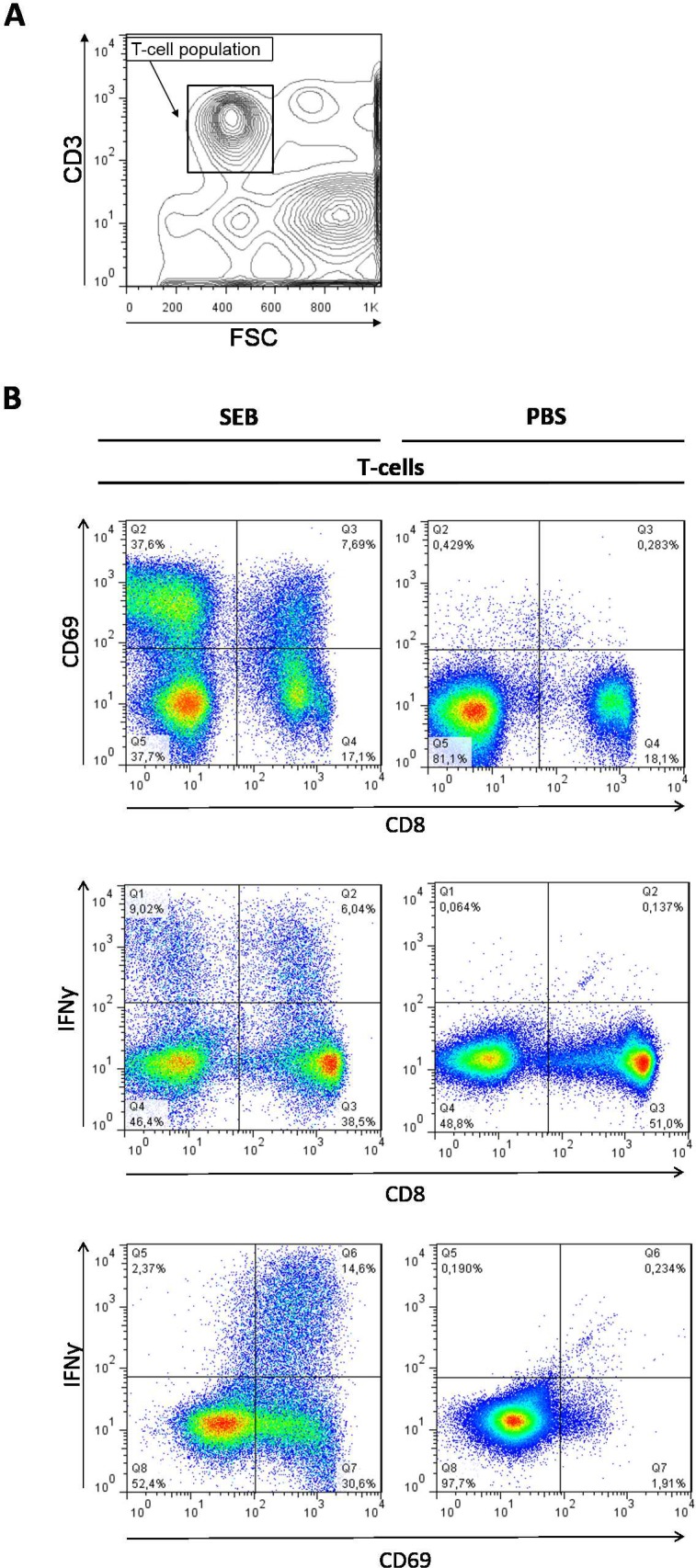
Representative example and gating strategy for assessment of T-cell subpopulations. A: Gating on CD3-positive cells. B: Analysis of expression of CD69 on the surface and intracellular IFNγ of CD8-positive T-cells (CD3) upon SEB- and PBS-stimulation of a whole blood sample from a healthy control. 100,000 CD3-positive events were analysed.

The gating strategy and a representative example of the flow cytometry data are presented in Fig 1 and correspond to a previously described procedure [18] with examination of subpopulations of activated (CD69-positive) and IFNγ-producing T-cells (CD3-positive).

For each measurement of SEB- and CMVpp52-stimulated samples, the result obtained from the negative control sample with PBS stimulation was subtracted. A sample was considered positive for activated T-cells if the percentage of CD69-positive T-cells was higher than 0.98% (cut-off value determined by the mean value of all measurements), and was considered to have a high number of activated T-cells if the percentage of CD69-positive T-cells was higher than 4.09% (high cut-off value determined by mean + 2xSD).

### Cytokine quantification by multiplexed Luminex assay

The concentrations of five cytokines (IFNγ, IL10, IL12, IL17, and TNFα) secreted upon stimulation with CMVpp52, HHV6p41, SEB, PBS, and β-galactosidase were analyzed with an in-house assay by multiplexed Luminex xMAP technology (Luminex Corp, TX, USA) as previously described [30, 32].

In short, two 3 mm diameter disks were punched from each DBSS of stimulated whole blood and then extracted. Next, the extracted samples were incubated with capture antibody-conjugated beads. After washing, the samples were incubated first with corresponding biotinylated detection antibodies and then with streptavidin-PE. After additional washing, samples were analyzed on the Luminex 100^TM^ platform (Luminex Corp, TX, USA).

In addition to the standard assay conditions, heterophillic blocking reagent plus (HBR+) (3KC545 Scantibodies Laboratory, Inc., Santee, CA, USA) was added to samples (from both SLE and HC) and conjugate in a concentration of 400 μg/ml to avoid false positive results due to rheumatoid factors/heterophilic antibodies.

The working ranges were 8–4000 pg/ml for IL12 and IL17; and 156–80000 pg/ml for IL10, TNFα, and IFNγ. All samples from each SLE/HC-pair were always analyzed on the same plate, and each plate contained a high and a low positive control together with the standard curve. The standard curves were fitted with a five-parameter logistic equation (Logistic-5PL) using BioPlex^TM^ Manager 6.1 (Bio-Rad Laboratories, CA, USA).

### Detection of antibodies in plasma by ELISA

Specific CMVpp52-directed antibodies were detected in plasma from included individuals (the 17 SLE patients and 17 HCs included for flow cytometric T-cell analysis) by the use of an ELISA setup previously employed [22]. Briefly, Nunc polysorp microtitre plates (Thermo Fisher Scientific, Denmark) were coated with CMVpp52 (1 **μg**/ml, *Escherichia coli* (*E-coli*)-derived, CMV-214, Prospec Protein Specialist, Ness-Ziona, Israel) in carbonate buffer (50 mM sodium carbonate, pH 9.6) over night at 4°C. After washing, plates were blocked for 30 minutes in TTN buffer (0.05 M Tris, 1% Tween20, 0.3 M NaCl, pH 7.5). Plasma samples were diluted 1:100/1:100/1:50 for detection of CMVpp52-directed IgM/IgG/IgA antibodies, respectively. Diluted samples were added in duplicates to both coated and non-coated wells. After washing, the plates were incubated with alkaline phosphatase (AP)-conjugated goat anti-human IgM, IgG or IgA (Sigma-Aldrich, St. Louis, MO, USA) (1:2000). Plates were developed after extensive washing by adding ρ-nitrophenol phosphate (ρ-NPP) (Sigma-Aldrich, St. Louis, MO, USA) in substrate buffer (1M diethanolamine, 9.5 mM MgCl_2_, pH 9.8) (1 mg/ml). Plates were read by a Versamax microplate reader (Molecular Devices, Sunnyvale, CA, USA) with a wavelength of 405 nm and a reference wavelength of 650 nm.

The absorbance values from the non-coated wells were subtracted from the absorbance values from the coated wells and all net absorbance values were then normalized to standard curves derived from a two fold serial dilution of serum pools. A plasma sample was considered positive for CMVpp52-antibodies if the antibody binding was higher than cut-off values (0.5, 0.64 and 0.5 arbitrary binding units regarding CMVpp52-directed IgM, IgG and IgA, respectively).

Total CMV antibodies were determined as previously described using a cellular CMV lysate as antigen and cellular lysate without CMV as control [33, 34].

### Statistical analyses

Statistical analyses were made in GraphPad Prism software 5 (GraphPad Prism Software Inc, San Diego, CA, USA). Comparison between SLE patients’ and HCs’ T-cell responses (CD69-positives and intracellular IFNγ) measured by flow cytometry, and also cytokine responses measured by Luminex technology were performed using Wilcoxon matched-pairs test. Spearman’s correlation test was used for correlation analyses. Fisher’s exact test was used for comparisons on SLE patients and HCs divided in positives and negatives for CMVpp52-responding T-cells and CMVpp52-directed antibodies, respectively. p-values below 0.05 were considered significant and indicated on Figs and tables with *, **, or *** for p-values less than 0.05, 0.01, or 0.001, respectively.

To be able to compare the current results to previously published results on flow cytometric analyses of EBV-responding T-cells [18] and EBV-induced cytokines quantified by Luminex technology [30], results are presented here as in the preceding papers with mean±SEM for flow cytometric T-cell analyses data, and median with interquartile range for Luminex technology quantitative cytokine data.

## Results

In order to investigate the CMV-directed immune response in SLE patients and compare it to the normal response in HCs, the CMVpp52-directed T-cell response was characterized, and its relation to the CMVpp52 antibody status was evaluated. Furthermore, CMVpp52- and also HHV6p41-induced cytokines were examined.

### Reduced T-cell response in SLE patients upon CMV stimulation

A 4-color flow cytometric T-cell analysis was performed to characterize T-cells upon stimulation of whole blood samples from 17 SLE patients and 17 HCs with the lytic cycle CMV antigen pp52, in order to investigate whether the previously established reduced T-cell response to EBV antigens [18] also applies to the CMV-directed T-cell response.

**Fig 2:**
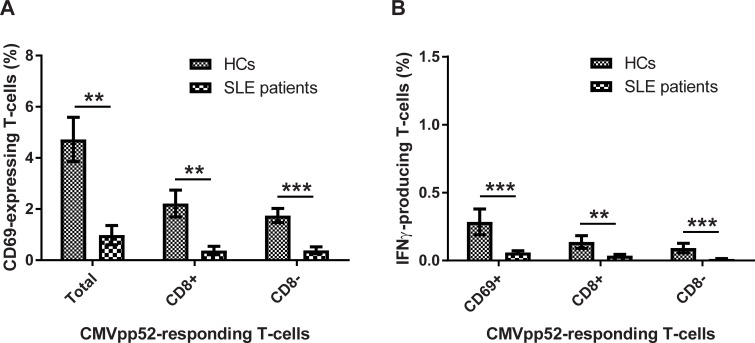
**Flow cytometric data on activated (A) and IFNγ-producing (B) T cells in SLE patients and HCs upon CMVpp52 antigen stimulation.** Heparinised whole blood samples from SLE patients (n = 17) and age- and sex-matched HCs (n = 17) were stimulated with CMVpp52. Data are presented as mean±SEM. Comparisons of T-cell responses between SLE patients and HCs were performed using Wilcoxon matched-pairs test (p-values in brackets). **A:** The percentages of activated T-cells in SLE patients and HCs separated in the total number of CD69-expressing T-cells (p = 0.001), and CD8+ (p = 0.004), and CD8- (p = 0.0004) T-cells expressing CD69, respectively. **B:** The percentages of IFNγ-producing T-cells separated in CD69-expressing T-cells (p = 0.0007), and CD8+ (p = 0.003), and CD8- (p = 0.0009) T-cells producing IFNγ, respectively.

Activated T-cells, and T-cells that produced IFNγ, upon CMVpp52 stimulation were determined and control stimulation values with PBS were subtracted. Fig 2 illustrates the percentages of T-cells (CD3-positives) from SLE patients and HCs that express CD69 on their surface (activated T-cells) (Fig 2A), and/or were producing intracellular IFNγ (Fig 2B) in response to CMVpp52 stimulation. Results show a statistically significant reduced percentage of CMVpp52-responding T-cells in SLE patients compared to HCs. The SLE patients experienced a weaker CD69 expression on the surface with statistically significant fewer T-cells, both CD8+ and CD8-, becoming activated (p = 0.001, 0.004, and 0.0004, regarding total, CD8+, and CD8-, CD69-expressing T-cells upon CMVpp52 stimulation) (Fig 2A). In addition, the SLE patients experienced statistically significant reduced percentages of T-cells, both CD8+ and CD8-, producing IFNγ upon CMVpp52 stimulation compared to HCs (p = 0.0007, 0.003, and 0.0009, regarding CD69, CD8+, and CD8-, IFNγ-producing T-cells upon CMVpp52 stimulation), however, with low percentages of IFNγ-producing T-cells in both SLE patients and HCs (Fig 2B).

As observed in the previous study on EBV antigen-responding T-cells [18], no statistically significant differences between SLE patients and HCs were observed upon stimulation with the superantigen SEB. However, with an exception of IFNγ-producing CD8- T-cells, with SLE patients having a significant (p = 0.033) lower percentage of IFNγ-producing CD8- T-cells compared to HCs upon SEB stimulation. However, these percentages of IFNγ-producing T-cells are all very low (especially for CD8- T-cells), and should be interpreted cautiously regarding all stimulations.

**Fig 3:**
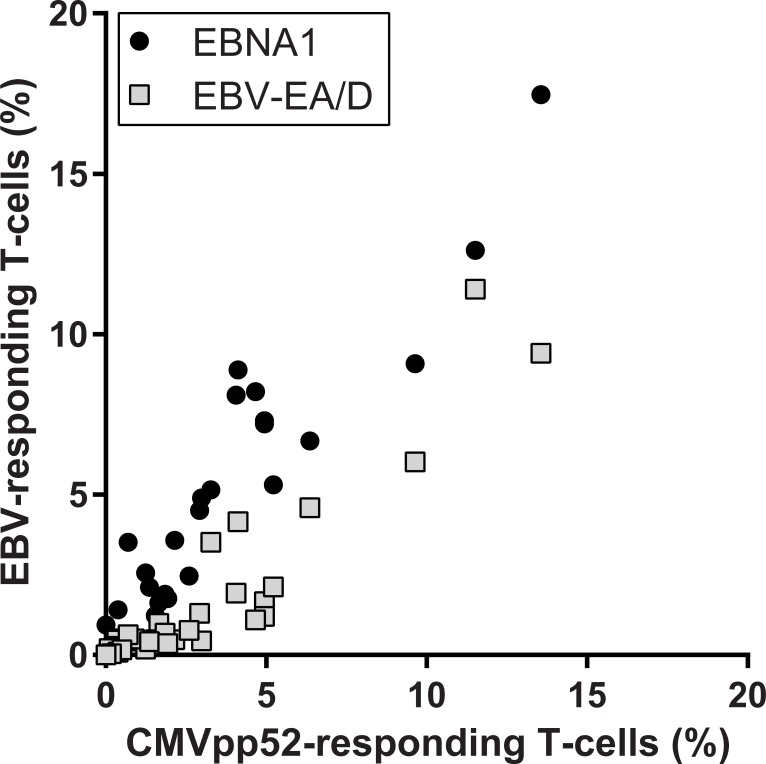
Correlation analyses of associations between percentages of CMVpp52-responding and EBNA1- or EBV-EA/D-responding T-cells, respectively (CD69-expressing T-cells upon antigen stimulation). Spearman’s correlation coefficients (r) are 0.933 (p<0.0001) and 0.917 (p<0.0001) when correlating percentages of CMVpp52-specific T-cells with EBNA1- (●) or EBV-EA/D-responding (□) T-cells, respectively.

Comparisons of the results in Fig 2 on CMVpp52-responding T-cells and previous results on EBV-responding T-cells are illustrated in Fig 3 and show a strong correlation to both EBNA1-responding T-cells (r = 0.933, p<0.0001), and EA/D-responding T-cells (r = 0.917, p<0.0001), indicating an association between the viral responses, which was valid for both SLE patients and HCs.

No correlation was observed between CMVpp52-responding T-cells and disease activity (determined by the SLEDAI score) or intake of immunosuppressant medication of the SLE patients.

### Outline of T-cell responses and antibody responses against CMV

The antibody status against CMVpp52 was determined by indirect ELISA in plasma from unstimulated blood samples from the SLE patients and HCs also included in the flow cytometric assay above. The results are listed in Table 2 and Table 3. In the previous investigations on EBV-directed immune responses [18], a clear inverse relation between EBV-responding T-cells and EBV-directed antibodies was revealed, and it was investigated if the same pattern emerged for the CMV-directed immune response.

**Table 2:** CMVpp52 antibodies and CMVpp52-specific T-cells in SLE patients and healthy controls.

	CMVpp52-specific (CD69) T-cells				CMVpp52 antibodies		
No.	Total	CD8+	CD8-		IgG	IgM	IgA
SLE-07							
SLE-09							
SLE-10							
SLE-15							
SLE-18							
SLE-20							
SLE-08							
SLE-19							
SLE-06							
SLE-26							
SLE-13							
SLE-12							
SLE-252							
SLE-17							
SLE-14							
SLE-27							
SLE-28							
							
HC-07							
HC-09							
HC-10							
HC-15							
HC-18							
HC-20							
HC-08							
HC-19							
HC-06							
HC-26							
HC-13							
HC-12							
HC-252							
HC-17							
HC-14							
HC-27							
HC-28							
	**Color code**						
	**CMVpp52-specific (CD69+) T-cells**				**CMVpp52 antibodies**		
	>4.09%	>0.98%	<0.98%		>2*cutoff	>cutoff	<cutoff
							

**Table 3:** Overview of CMVpp52 antibody status and CMVpp52-specific T-cells in SLE patients and healthy controls.

	CMVpp52-specific (CD69) T-cells			CMVpp52 antibodies		
	Total	CD8+	CD8-	IgG	IgM	IgA
SLE patients (n = 17), %	24	12	18	35	35	59
Healthy controls (n = 17), %	100	82	88	18	47	29
Comparison by Fisher’s exact test, p-values	<0.0001	<0.0001	<0.0001	0.44	0.73	0.17

In Table 2, an outline of results on both CMVpp52-responding T-cells (CD69-expressing T-cells upon stimulation) and CMVpp52-directed antibodies for each included individual are presented with color codes. The colors dark green, medium green, and light green represent positive for high numbers of activated T-cells (above the high cut off value), positive for activated T-cells (above the cut off value), and negative for activated T-cells (below the cut off value), respectively, regarding T-cell responses. Regarding antibody status, the colors represent positive for high titres of antibodies (above 2*cut off value), positive for antibodies (above the cut off value), and negative for antibodies (below the cut off value), respectively. SLE patients are listed with increasing disease activity in Table 2 (determined by the SLEDAI scores) (and the HCs are listed according to their matched SLE patients).

Table 2 clearly outlines the lower CMVpp52-directed T-cell response in the SLE patients compared to the HCs shown above (Fig 2A) but on the individual level. Furthermore, it shows a tendency of higher titres of CMVpp52-directed antibodies in SLE patients compared to HCs, though no statistical test was employed. However, the increasing disease activity do not seem to have an impact on the CMVpp52-directed immune responses in the SLE patients (which the above rejected correlation test between T-cell responses and SLEDAI scores also showed).

The pattern with the inverse relation previously demonstrated for the EBV-directed response with HCs having high percentages of virus-responding T-cells and few antibodies, and SLE patients having very few T-cells but high titres of antibodies [18], is also indicated for the CMVpp52-directed response in Table 2. However, to a lesser extent than demonstrated with the EBV-directed response.

Table 3 provides an overview of percentages of SLE patients and HCs positive for CMVpp52-responding T-cells (with CD69-expressing T-cells above the cut off value upon CMVpp52-stimulation) and CMVpp52-directed antibodies (titres above cut off values). Results on CMVpp52 antibody status show that higher percentages of SLE patients were positive for IgG and IgA with positivity percentages of 35% and 59% compared to 18% and 29% of HCs, respectively (Table 3). Contrarily, a slightly lower positivity percentage of IgM CMVpp52-directed antibodies was observed in SLE patients (35%) compared to HCs (47%) (Table 3). However, Fisher’s exact test showed no significant differences in antibody-positivity between SLE patients and HCs. The strong differentiation between SLE patients and HCs in percentages of CMVpp52-responding T-cells regarding both total number of CD69-expressing T-cells, and CD8+ and CD8- T-cells expressing CD69, was further outlined in Table 3. All 17 HCs were found positive for CMVpp52-responding T-cells compared to only 4 (24%) of the 17 SLE patients. Fisher’s exact test showed significant differences in positivity of CMVpp52-responding T-cells between SLE patients and HCs (p<0.0001), regarding both total, CD8+, and CD8- T-cells, respectively, as expected confirming results in Fig 2.

All of the 17 HCs and 14 of the 17 SLE patients showed either CMVpp52-directed antibodies and/or CMVpp52-responding T-cells, indicating positive CMV immune status. The last three SLE patients (negative in our initial analyses), were all found to have positive CMV serology measuring the total CMV antibody titer. Thus, the above presented results do not reflect a distortion in previous CMV infection among the SLE patients and HCs.

### CMV- and HHV6-induced cytokine responses

The cytokine responses (IFNγ, IL12, IL17, TNFα, and IL10) against CMV and HHV6 were characterized in SLE patients and compared to HCs by quantifying cytokines secreted upon stimulation with the early lytic cycle antigens CMVpp52 and HHV6p41. As previously applied (with results on EBV-induced cytokines [30]) in order to assess the issue without an effect on results due to lymphopenia (which is common in SLE patients), only SLE patients with normal lymphocyte levels (>1.00*10^9^ /L) were compared to their corresponding matched HC in the following results.

Unlike previous results on EBV antigen-induced cytokines, no difference in any of the quantified secreted cytokines were observed between SLE patients and HCs upon CMVpp52-stimulation (Table 4). Both SLE patients and HCs had a significant induction of all cytokines (except IL17 in SLE patients) by stimulation with CMVpp52 compared to PBS stimulation and thus to normal levels.

**Table 4:** Cytokine responses in SLE patients and HCs upon HHV-stimulation. Median concentration (pg/ml) [interquartile range].

	CMVpp52-induced cytokines			HHV6p41-induced cytokines		
Cytokine	HCs (n = 17)	SLE patients (n = 17)	p-value	HCs (n = 8)	SLE patients (n = 8)	p-value
IFNγ	3736 [1946–6564]	1239 [303–5121]	0.27	2387 [410–12979]	78 [78–2266]	0.22
IL12	306 [182–352]	252 [137–337]	0.91	299 [222–390]	223 [24–324]	0.03*
IL17	242 [42–452]	209 [4–377]	0.27	291 [38–374]	327 [58–352]	0.84
TNFα	18229 [14661–24486]	21042 [13413–24918]	0.78	20569 [13523–20838]	16928 [11486–24458]	0.55
IL10	21902 [21149–25792]	21738 [17923–24500]	0.30	19391 [17613–22042]	19400 [16510–20439]	0.64

All cytokine responses were compared using Wilcoxon matched-pairs test

The same pattern was observed after stimulation with HHV6p41 (however only eight SLE/HC-pairs were investigated making results less conclusive) with nearly no difference in cytokine concentrations between SLE patients and HCs (Table 4). Only IL12 induction was significantly different between the groups, but looking at the similar medians (Table 4) this difference is probably negligible.

No differences were observed between SLE patients and HCs upon PBS stimulation (served as basic levels of cytokines) in any of the 5 quantified cytokines as also demonstrated in the previous study.

Actually, even though no differences between SLE patients and HCs were observed in cytokine secretions upon β-galactosidase stimulation (which served as a control for antigens produced in *E*.*coli*), an induction was observed in IL10 secretion upon stimulation with β-galactosidase compared to PBS stimulation in both SLE patients (p = 0.008) and HCs (p = 0.03). This might make the results on CMVpp52- and HHV6p41-induced cytokines inconclusive, as the antigens are also produced in *E*.*coli*. However, the induction of IL10 was significantly higher in HHV-stimulated samples compared to β-galactosidase-stimulated samples. This result, together with the fact that the β-galactosidase induction is similar between SLE patients and HCs, indicates that the results on a normal CMVpp52- and HHV6p41-induced cytokine response in SLE patients should be valid.

Statistically significantly reduced secretion of IFNγ, IL17, and TNFα was observed in SLE patients compared to HCs upon stimulation with the superantigen SEB (Table 5). Due to exclusion of SLE patients with lymphopenia, these results might be an effect of dysfunctional leukocytes in SLE patients.

**Table 5:** Cytokine responses in SLE patients and HCs upon SEB-stimulation. Median concentration (pg/ml) [interquartile range].

	SEB-induced cytokines			PBS-induced cytokines (basic levels)		
Cytokine	HCs (n = 17)	SLE patients (n = 17)	p-value	HCs (n = 17)	SLE patients (n = 17)	p-value
IFNγ	28950 [23178–42766]	16547 [11904–25201]	0.003**	78 [78–200]	78 [78–78]	0.31
IL12	264 [183–356]	230 [177–336]	0.43	67 [4–180]	95 [4–168]	0.90
IL17	2384 [1465–4000]	1922 [1475–2152]	0.04*	4 [4–238]	4 [4–310]	0.49
TNFα	16326 [11355–18373]	10166 [6139–12807]	0.001**	78 [78–489]	78 [78–78]	0.69
IL10	9457 [5658–12373]	7920 [5327–10582]	0.37	1544 [787–3913]	1920 [1151–3456]	0.92

All cytokine responses were compared using Wilcoxon matched-pairs test

In order to check if the above presented results on CMV-directed cytokine-responses is due to a distortion between SLE patients and HCs in CMV immune status, total CMV antibody titers were also measured in the individuals. Actually, results showed that three of the 17 SLE patients and four of the 17 HCs in this cohort 2 were negative for CMV serology. Exclusion of the data from these individuals revealed no differende in outcome with still no significant differences in cytokine responses between SLE patients and HCs. This indicates that the above presented results do not reflect a distortion in previous CMV infection among SLE patients and HCs.

## Discussion

In this study, we have presented an outline of immune responses to the early lytic CMV antigen, CMVpp52, in SLE patients. This comprised results on percentages of CMVpp52-reponding T-cells by flow cytometry and comparison to CMVpp52 antibody status measured by ELISA, and also CMVpp52-induced cytokine response patterns quantified by multiplexed Luminex technology. Furthermore, cytokine responses to its functional homologue for HHV6, HHV6p41, were briefly studied.

We sought to clarify if the previously determined reduced T-cell response and cytokine response pattern in SLE patients upon EBV antigens stimulations [18, 30] is specific for EBV, or if SLE patients have a general immune defect regarding responses to more HHVs. EBV-EA/D, CMVpp52, and HHV6p41 are all early antigens of their respective HHVs, and serves as DNA polymerase processivity factors and are essential for the productive cycle of the viruses [35–38]. Investigations of immune responses against these functional homologues of three HHVs in SLE patients make direct comparisons possible in the evaluation of the immune defects in SLE patients and whether it comprises all three HHVs or is specific for EBV responses.

Overall, results were diverse with decreased percentages of responding T-cells upon CMVpp52-stimulation but with normal cytokine response patterns to both CMVpp52 and HHV6p41 in SLE patients compared to HCs.

Regarding, flow cytometric results investigating activation of the individual cells, the CMVpp52 response seemed decreased with statistically significantly fewer T-cells becoming activated in SLE patients compared to HCs, even though a rather small sample size was employed (n = 17). These findings correlated with earlier results on EBV-responding T-cells, but did not correlate with disease activity of the SLE patients. Furthermore, a higher positivity of IgG and IgA antibodies against CMVpp52 were detected in SLE patients.

This observed reduced CMV-specific T-cell response is somewhat contradictory to previous observations in SLE patients [26, 27]. Larsen et al. applied MHCI tetramers with CMVpp65 peptides, and PBMCs from SLE patients (n = 21) and HCs (n = 15), and showed normal amounts of CD8 CMVpp65-specific T-cells in SLE patients with normal cytokine responses upon CMV stimulation [26]. Kang et al. stimulated whole blood samples for six hours with unspecified CMV antigens and only found a tendency of a reduced CMV-directed T-cell response in SLE patients (n = 17) compared to HCs (n = 9) [27]. In the current study, the percentages of functional CD8+ and CD8- lytic antigen CMVpp52-responding T-cells was found reduced in SLE patients, which is opposite of results by Larsen et al and Kang et al. However, the differences might be due to the use of different CMV antigens (pp65 as opposed to pp52) and also differences in experimental setups (PBMCs and MHCI tetramers as opposed to whole blood samples with various stimulation times), and cohorts which are all relatively small and might represent different subgroups of patients.

Our current results on reduced percentages of CMVpp52-responding T-cells are possibly a consequence of limited or defective CMVpp52-specific T-cells. As discussed previously with the reduced percentages of EBV-responding T-cells, a possible reason is hyperactivation and subsequent exhaustion of HHV-specific T-cells in SLE patients upon continuous exposure to HHV antigens following frequent and recurrent HHV reactivation, a theory also proposed by Larsen et al. [18, 26].

However, due to the chosen methodology comprising whole blood stimulation, another possibility is that the decreased T-cell activation observed in SLE patients is not due to intrinsic T-cell defects, but rather indirect effects on T-cell activation, since T-cell activation is complex with multiple influential factors. Previous studies on SLE patients show altered cytokine levels in the circulation [39–41] giving rise to an altered, and perhaps suboptimal environment for T-cell activation in SLE patients compared to HCs. The most characteristic observation in this regard is the IFN signature in SLE patients with increased levels of type I IFNs, especially IFNα [42], which is actually known to reduce the T-cell response during chronic infections and inflammation [43]. Another indirect effect, possibly leading to decreased T-cell activation is poor antigen presentation, which is of special importance in current experimental setup using whole blood samples and stimulation with exogenous antigens. Previous studies have pointed to defects in antigen presentation in SLE patients [44, 45], which would influence the T-cell activation. Since Larsen et al. found normal levels of CMV-specific T-cells in SLE patients with normal effector responses using CMV-MHCI tetramers [26], our results on decreased T-cell activation upon CMVpp52 stimulation possibly reflect poor antigen presentation in the SLE patients.

As previously discussed, a general limitation and challenge in the current study is the experimental approach, and the process of activation of CD8 T-cells upon stimulation with an exogenous antigen. Previous studies suggest a cross-presentation pathway of exogenous antigens [46–49] or neighboring bystander activation but the exact reason for the observed activation of CD8 T-cells is not clear. Nonetheless, this experimental setup makes it difficult to evaluate the exact mechanisms of the immune stimulation including antigen processing and the T-cell response. Thus, the interpretation of the underlying reasons for the data presented in this study is difficult to assess.

The previously shown pattern of an inverse relation for the EBV-directed immune response with SLE patients having low percentages of EBV-responding T-cells and high amounts of antibodies, and the reverse for HCs [18], was also proposed in the current study for the CMVpp52-directed immune response, however, to a much lesser extent. As previously discussed, it could be speculated that the deficient T-cell response to HHVs (and thereby probably a deficient control of HHV infections), may cause a shift in the immune reaction towards a humoral response in order to compensate for the lacking cell-mediated response. Another reason might be reduced HHV antigen removal subsequent to recurrent reactivations. Irrespective of the function, the observed higher positivity percentages of IgG and IgA antibodies against CMVpp52 in SLE patients further indicates a defect in the CMV-directed immune response, which also was previously shown by Rasmussen et al. with elevated titres of CMVpp52-directed antibodies in another SLE cohort [22]. A future study on cell-associated CMV loads in SLE patients would clarify if the immune reaction against CMV is exhausted in SLE patients and the T-cell control of CMV is dysfunctional and therefore results in CMV reactivations and increased viral load. This would also explain the increased lytic cycle antigen CMVpp52-directed antibody titres in SLE patients.

As no correlation was observed between lytic early antigen CMVpp52-responding T-cells and disease activity of the SLE patients, (which previously was demonstrated with the EBV functional homologue lytic early antigen EBV-EA/D-responding T-cells [18]), the reduced CMVpp52-responding T-cells might not be involved in the exacerbation of SLE to the same extent as we previously have proposed for the reduced EBV-responding T-cells. However, interestingly, the results on CMVpp52-responding T-cells correlated with the earlier results on EBNA1- and EBV-EA/D-responding T-cells [18], which suggested some relation of the viral immune responses. Results propose a similar reaction (or lack of) to the HHV infections in the individuals. These results indicate that the poor control of one virus is reflected in poor control of other HHVs and possibly other viral infections.

The second setup on Luminex-quantified cytokines induced by CMVpp52 (and also HHV6p41) were contradictive to the observed reduced percentages of CMVpp52-responding T-cells by flow cytometry.

Our previous results on cytokines induced by EBV antigens showed that the SLE patients had significantly lower cytokine secretions than HCs regarding numerous T-cell- and/or NK-cell-related cytokines [30]. But here we showed that this presumably is specific for the response to EBV antigens, as stimulation with both CMVpp52 or HHV6p41 induced a normal cytokine response pattern in SLE patients regarding both T-cell response-related cytokines (IL12, IFNγ, (IL17)) and also both inflammatory (TNFα, IL17) and anti-inflammatory (IL10) cytokines. However, the small sample size investigated in this study and the chosen methodology comprising whole blood stimulation (and possibly poor antigen-presentation to follow) designate that these results should be interpreted lightly.

The normal basic levels of cytokines in SLE patients (as also seen in the previous study, [30]) indicated that the SLE patients without lymphopenia actually have a normal distribution of cytokines without aberrant exaggerated immune stimulation. As previously stated, this ensures that any differences in induction of cytokines upon CMVpp52 and HHV6p41 stimulation are not due to differences in basic levels between the groups.

Stimulation with the negative control, *E*.*coli*-derived β-galactosidase actually induced production of IL10 compared to stimulation with PBS in both SLE patients and HCs, but the induction was significantly lower than by stimulation with HHV antigens, and furthermore, it was similar between the groups. These results are probably not a general effect of LPS from the *E*.*coli* production as not all quantified cytokines were induced precipitously upon β-galactosidase stimulation.

The significant difference between SLE patients and HCs observed upon stimulation with the superantigen SEB, regarding IFNγ, IL17, and TNFα may indicate that leukocytes are generally dysfunctional in the SLE patients, also in those without lymphopenia, and unable to respond properly to the stimuli. The dysfunctional leukocytes presumably comprise macrophages and Th17 cells not able to uphold the inflammatory response to SEB, and perhaps also T-cells and/or NK-cells unable to activate the cell-mediated immune response. Low cytokine responses to SEB in SLE patients were also demonstrated in the previous cytokine secretion study regarding EBV [30]. Contrary to this though, is that this was not suggested by the flow cytometric analysis of CMVpp52-responding T-cells where SEB stimulation showed normal responses in SLE patients.

Overall, the contradictive nature of the current results on CMVpp52-directed immune responses in SLE patients with decreased activity of T-cells in the flow cytometric analysis but with normal cytokine-induction measured by Luminex technology could have several reasons. One might be the fact that different SLE patient and HC cohorts were employed in the two setups (with a difference in mean disease activities between the cohorts), or the fact that flow cytometric results are normalized as we are investigating percentages of T-cells, and cytokine results are actual values and not normalized to number of cells or other factors. Another reason might be that the clear dysfunctional immune responses against EBV observed in multiple studies on SLE patients do not apply to the same degree regarding the immune responses against CMV or HHV6. This might be due to the variant but overlapping tropisms of EBV, CMV and HHV6, and thereby differences in infected cells involvement in SLE according to the individual patient’s immune profiles. Elevated titres of antibodies to EBV-EA/D and CMVpp52 but not to HHV6p41 have previously been reported in SLE patients by Rasmusssen et al. [22]. Perhaps, the main viral contributing agent to the development or exacerbation of SLE in genetically predisposed individuals is the uncontrolled EBV infection, and to a lesser extent CMV infection and probably with no involvement of HHV6 infection. In fact it cannot be ruled out that the observed reduced CMV-induced T-cell activation is an effect of poor antigen presentation in SLE patients.
